# Effect of hydrocortisone on the 28-day mortality of patients with septic acute kidney injury

**DOI:** 10.1080/0886022X.2019.1658605

**Published:** 2019-09-05

**Authors:** Pan Ying, Chenguang Yang, Xianlong Wu, Qiqi Cai, Wenwei Xin

**Affiliations:** a Taizhou First People's Hospital, Taizhou, China;; b Health Care Center for Women and Children of Huangyan District, Taizhou, China

**Keywords:** Hydrocortisone, mortality, sepsis, acute kidney injury

## Abstract

**Objectives:** To evaluate the efficacy of hydrocortisone in patients with septic acute kidney injury (SAKI).

**Methods:** This retrospective cohort study consisted of all consecutive patients with SAKI who were admitted to the Taizhou First People's Hospital from March 2016 to February 2018. The patients who were treated with usual care including antibiotics, fluid resuscitation, and blood glucose control were regarded as the control group, and those received add-on hydrocortisone by the clinicians' discretion was considered in the intervention group. Hydrocortisone was administered as a 50 mg intravenous bolus every six hours for seven days. To adjust the potential baseline differences between the hydrocortisone and control groups, a 1:1 propensity score matching (PSM) was performed to identify a matched control subject for each patient in the hydrocortisone group.

**Results:** In the propensity-matched cohort, the 28-day mortality was significantly lower for patients in the hydrocortisone group (*p* = .04). Both Acute Physiology and Chronic Health Evaluation (APACHE) II and the Sequential Organ Failure Assessment (SOFA) scores were significantly lower at day 7 in the hydrocortisone group (both *p* < .01). Serum IL-1β, IL-6, and TNF-α concentrations significantly decreased for hydrocortisone group at day 7 (all *p* < .01). The levels of serum creatinine (SCr), Cystatin C (CysC), and procalcitonin (PCT) were significantly lower, while the levels of glomerular filtration rate (GFR) and urine volume were significantly higher for hydrocortisone group at day 7 (all *p* < .01).

**Conclusions:** Glucocorticoid supplementation may improve renal function and reduce the 28-day mortality of patients with SAKI.

## Introduction

Acute kidney disease (AKI) is a common and serious complication in patients with sepsis, affecting nearly two-thirds of patients admitted to intensive care units (ICUs) [1,2]. Especially, it occurred in 87.5% of patients with sepsis in a cohort study in Portugal [3]. The presence of AKI is associated with a poor prognosis in these patients, which increases patient morbidity, predicts higher mortality, and consumes considerable health resources. Sepsis is the most common contributing factor for the development of AKI. The Beginning, Ending Supportive Therapy for the Kidney (BEST Kidney), a large prospective observational study involving 29,000 patients reported that 47.5% of septic AKI (SAKI) accounted for all AKI cases [4]. In a cohort study in Portugal, AKI occurred in 87.5% of patients with sepsis.

Currently, the understanding of pathogenesis for SAKI is limited and treatment regimens including fluid, hemofiltration, diuretics, and antimicrobials have remained largely supportive. Despite treatment with multiple, newly developed therapeutic agents, the overall hospital mortality in patients with SAKI is still as high as 50–70% [4,5].

Experimental and clinical evidence suggests that sepsis is associated with a dysregulated response of the hypothalamic-pituitary-adrenal axis that may involve any of the steps from cortisol production to cortisol use by cells [6]. Glucocorticoids have been used as an adjuvant therapy for septic shock for more than 50 years and have been recommended by International Guidelines for Management of Severe Sepsis and Septic Shock. Two large-scale randomized controlled trials (ADRENAL [7] and APROCCHSS [8]) reported in 2018 showed inconsistent results, only APROCCHSS showed that among 1241 patients with septic shock, 90-day all-cause mortality was lower among those who received hydrocortisone plus fludrocortisone than those received placebo. Nevertheless, both trials indicated that faster reversal of septic shock and shorter duration of mechanical ventilation in patients receiving hydrocortisone than in those receiving placebo. Another multicenter, placebo-controlled, randomized, double-blind study conducted by Laviolle et al. [9] indicated that low-dose hydrocortisone induced the glucocorticoid biological effects and seemed to improve renal function. SAKI is characterized by a distinct pathophysiology when compared with AKI of non-septic origin, thereby causing important differences in patient characteristics, response to interventions, and clinical outcomes [10,11]. Though glucocorticoids have been shown to attenuate SAKI in several animal models [12,13], its clinical effects are rarely reported. This retrospective cohort study aims to evaluate the efficacy of hydrocortisone in patients with SAKI.

## Methods

### Patients

This retrospective cohort study consisted of all consecutive patients with SAKI who were admitted to the Taizhou First People's Hospital from March 2016 to February 2018. Patients were eligible for enrollment if they were critically ill adults ≥18 years who had a medical record of SAKI. Each case of sepsis was defined according to The Third International Consensus Definitions for Sepsis and Septic Shock (Sepsis-3) [14], and AKI was diagnosed on the basis of Kidney Disease: Improving Global Outcomes (KDIGO) Clinical Practice Guideline for Acute Kidney Injury [15]. Patients met consensus criteria for both sepsis and AKI were deemed to have SAKI [16]. The stage of AKI was assessed by either creatinine or urine output criteria [17]. Patients with life expectancy less than 3 months, terminal cancer, history of dialysis or pregnant were excluded.

The study was approved by the institutional review board of Taizhou First People's Hospital (KY2018-030-01) and conducted in accordance with provisions of the Declaration of Helsinki.

### Study design

The patients who were treated with usual care including antibiotics, fluid resuscitation, and blood glucose control were regarded as the control group, and those received add-on hydrocortisone by the clinicians' discretion were considered in the intervention group. Hydrocortisone was administered as a 50 mg intravenous bolus every 6 h for seven days.

### Study outcomes

The primary outcome was 28-day all-cause mortality. Secondary outcomes included the number of days that patients were alive up to day 28, 90-day all-cause mortality, the changes in the Acute Physiology and Chronic Health Evaluation (APACHE) II score and the Sequential Organ Failure Assessment (SOFA) scores, serum cytokine levels such as tumor necrosis factor-α (TNF-α), interleukin-1β (IL-1β), and IL-6, as well as renal functions including serum creatinine (SCr), Cystatin C (CysC), procalcitonin (PCT), glomerular filtration rate (GFR), and urine volume between admission to hospital and after seven-day treatment. CysC was measured by immunoturbidimetric assays. GFR was estimated using the CKD Epidemiology Collaboration (CKD-EPI) equation [18]. The PCT level was measured using an immunoluminometric assay, chemiluminescent enzyme immunometric assay.

### Statistical analysis

We determined that 95 patients each group would provide the study with 90% power to detect an absolute difference of 10 percentage points in 28-day mortality from an estimated baseline mortality of 45%, at an alpha level of 0.05.

To adjust for the potential baseline differences between the hydrocortisone and control groups, a 1:1 propensity score matching (PSM) was performed. Propensity scores were estimated using a probit model, controlling for all baseline characteristics. A matched control subject was identified for each patient in the hydrocortisone group using the nearest neighbor algorithm. After PSM, we assessed the quality of matching by comparing each baseline characteristics between two groups.

All quantitative data were expressed as mean ± standard deviation (SD) and compared by Student’s *t*-test. Categorical variables were presented as percentages and compared by Chi-square test and Fisher’s exact test. The log-rank test was used as the primary test of an overall difference between the Kaplan–Meier curves for survival. Cox’s proportional hazard model was used to perform multivariate analysis.

Statistical analysis and graph presentation were performed using SPSS version 20.0 software (SPSS Inc., Chicago, IL). All tests were two-tailed and a *p* value of <.05 was considered as statistically significant.

## Results

### Patients’ characteristics

A total of 653 adult patients with SAKI were screened, among which 228 had completed records and met the inclusion criteria. Of these patients, 133 received usual care and 95 (50 in the first year and 45 in the second year) received add-on hydrocortisone to usual care. A 1:1 PSM method was performed to identify 95 pairs of hydrocortisone add-on and control patients who had similar baseline characteristics and biochemical variables (Table 1). The use of renal replacement treatment during the hospitalization was also not significantly different between two groups.

**Table 1.** Baseline characteristics of patients with septic acute kidney injury in hydrocortisone group and control group.

	Hydrocortisone group (*n* = 95)	Control group (*n* = 95)	*p* Value
Age, years	60.8 ± 14.8	60.5 ± 14.9	.89
Male, *N* (%)	53 (55.8%)	52 (54.7%)	1.00
Body mass index, kg/m^2^	23.5 ± 4.5	23.3 ± 4.7	.77
Systolic blood pressure, mm Hg	106 ± 27	107 ± 28	.80
Stage of acute kidney injury			
1	34 (35.8%)	31 (32.6%)	.14
2–3	61 (64.2%)	64 (67.4%)	
Comorbidities, *N* (%)			
Diabetes mellitus	27 (28.4%)	26 (27.4%)	1.00
Hypertension	55 (57.9%)	57 (60.0%)	.88
Chronic respiratory disease	24 (25.3%)	24 (25.3%)	1.00
Cancer	17 (17.9%)	18 (18.9%)	1.00
Heart Failure			
Cerebral vascular disease	8 (8.4%)	7 (7.4%)	1.00
Peripheral vascular disease	7 (7.4%)	7 (7.4%)	1.00
Hepatic cirrhosis	7 (7.4%)	6 (6.3%)	1.00
Type of infection, *N* (%)			
Gastrointestinal	33 (34.7%)	31 (32.6%)	.88
Respiratory	40 (42.1%)	41 (43.2%)	1.00
Genitourinary	6 (6.3%)	7 (7.3%)	1.00
Neurologic	4 (4.2%)	4 (4.2%)	1.00
Others	14 (14.7%)	12 (12.6%)	.83
APACHE II score	22.7 ± 4.8	22.5 ± 4.9	.78
SOFA score	10.8 ± 2.1	10.9 ± 2.2	.75
Serum cytokine levels			
IL-1β, pg/mL	2.5 ± 1.4	2.6 ± 1.8	.48
IL-6, pg/mL	518.4 ± 217.5	491.7 ± 200.3	.79
TNF-α, pg/mL	58.2 ± 27.4	56.3 ± 25.1	.57
Renal function			
SCr, μmol/L	3.1 ± 2.7	3.2 ± 2.8	.80
CysC, mg/dL	2.5 ± 0.5	2.6 ± 0.4	.13
GFR, mL/min	61.4 ± 9.1	63.2 ± 10.2	.20
PCT, μg/L	13.8 ± 3.2	14.1 ± 2.8	.49
Urine volume, mL/d	657.3 ± 111.2	631.4 ± 107.8	.10
Renal replacement therapy use during hospitalization	5 (5.3%)	7 (7.4%)	.77

### Primary outcome

At day 28, death had occurred in 30 of 95 patients (31.6%) in the hydrocortisone group and in 44 of 95 patients (46.3%) in the control group. The relative risk of death was 0.72 (95% CI, 0.53–0.99) in favor of hydrocortisone therapy.

### Secondary outcomes

**Figure 1.**
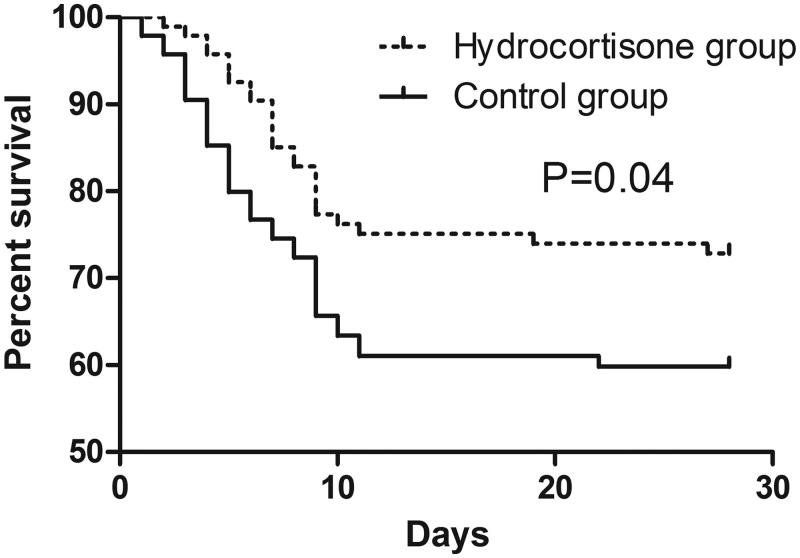
The survival curves of patients in the hydrocortisone group and control group.

The survival curves of both groups are presented in Figure 1. The log-rank test showed that the survival was significantly longer for patients in the hydrocortisone group compared with that in the control group (HR = 0.59; 95% CI: 0.35–0.98; *p*= .04). After adjusting for all baseline characteristics, multivariate analysis illustrated that hydrocortisone (HR = 0.73; 95% CI: 0.51–0.84; *p* < .01) was negatively associated with the mortality, while APACHE II (HR = 1.14; 95% CI: 1.05–1.26; *p*= .03) and SOFA (HR = 1.39; 95% CI: 1.17–1.69; *p* < .01) scores were positively associated with the mortality (Table 2).

**Table 2.** Independent predictors for 28-day mortality using the Cox proportional hazards model.

	Multivariate analysis		
	Hazard ratio	95% CI	*p* Value
APACHE II score	1.14	1.05–1.26	.03
SOFA score	1.39	1.17–1.69	<.01
Hydrocortisone vs. control group	0.73	0.51–0.84	<.01

The multivariate analysis was adjusted for all baseline variables.

**Figure 2.**
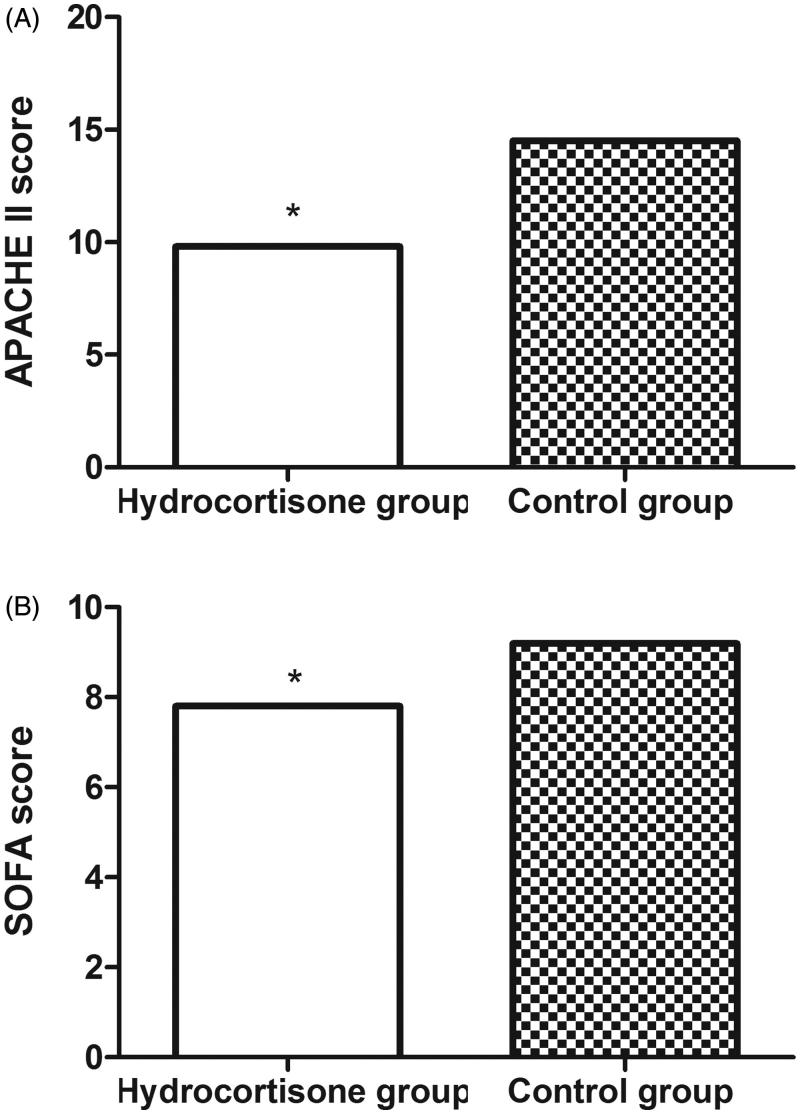
The APACHE II (A) and SOFA scores (B) of patients in the hydrocortisone group and control group at day 7. APACHE II: Acute Physiology and Chronic Health Evaluation II; SOFA: Sequential Organ Failure Assessment.

At day 7, both APACHE II score (9.8 ± 1.8 vs. 14.5 ± 2.1; *p* < .01) and SOFA score (7.8 ± 1.0 vs. 9.2 ± 1.5; *p* < .01) were significantly lower for patients in the hydrocortisone group than those in the control group (Figure 2).

As shown in Table 3, serum IL-1β (1.5 ± 1.0 vs. 1.9 ± 1.1; *p* < .01), IL-6 (162.5 ± 93.4 vs. 274.5 ± 101.2; *p* < .01), and TNF-α (25.4 ± 14.2 vs. 36.3 ± 23.1; *p* < .01) concentrations significantly decreased for hydrocortisone group compared with control group. The levels of SCr (0.9 ± 0.6 vs. 1.2 ± 0.5; *p* < .01), CysC (1.8 ± 0.3 vs. 2.5 ± 0.3; *p* < .01), and PCT (5.7 ± 1.5 vs. 8.9 ± 1.9; *p* < .01) were significantly lower, while the levels of GFR (104.8 ± 10.2 vs. 86.8 ± 9.8; *p* < .01) and urine volume (1028.4 ± 125.1 vs. 871.4 ± 120.5; *p* < .01) were significantly higher for hydrocortisone group, compared with control group.

**Table 3.** Comparisons of serum cytokine levels and renal functions at day 7 between patients in the hydrocortisone group and control group.

	Hydrocortisone group (*n* = 95)	Control group (*n* = 95)	*p* Value
Serum cytokine levels			
IL-1β, pg/mL	1.5 ± 1.0	1.9 ± 1.1	<.01
IL-6, pg/mL	162.5 ± 93.4	274.5 ± 101.2	<.01
TNF-α, pg/mL	25.4 ± 14.2	36.3 ± 23.1	<.01
Renal function			
SCr, μmol/L	0.9 ± 0.6	1.2 ± 0.5	<.01
CysC, mg/dL	1.8 ± 0.3	2.5 ± 0.3	<.01
GFR, mL/min	104.8 ± 10.2	86.8 ± 9.8	<.01
PCT, μg/L	5.7 ± 1.5	8.9 ± 1.9	<.01
Urine volume, mL/d	1028.4 ± 125.1	871.4 ± 120.5	<.01

## Discussion

Published studies mainly focused on the effects of glucocorticoids in patients with sepsis or patients with AKI [19–22], our study showed that glucocorticoid supplementation improved renal function and reduced the 28-day mortality of patients with septic AKI.

Sepsis-induced AKI seems to have a multifactorial pathogenesis, among which inflammation is regarded to play an important role in the disease progression. During sepsis, infection triggers a host response, in which inflammatory mechanisms contribute to clearance of infection and tissue recovery on the one hand, and organ injury on the other [23]. Previous studies demonstrated that repeated stimulation of the kidneys by sepsis led to the production of a large number of pro-inflammatory cytokines including TNF-α and IL-6 through nuclear factor-kappa B (NF-κB) signaling pathway, which is a key regulator in keeping homeostasis of immune system and a major drug target in a variety of diseases [24,25]. Our study indicated that the serum cytokines such as TNF-α, IL-1β, and IL-6 were inhibited by hydrocortisone, which were consistent with previous studies in both animals and humans with sepsis [26]. Moreover, recent evidence suggested that mitochondrial damage and subsequent defect in mitochondrial respiratory chain may be key factors in cellular dysfunction, leading to end-organ failure in sepsis. The glucocorticoids were demonstrated to provide organ protective effect by reducing mitochondrial injury with preserved cytochrome c oxidase and suppression of pro-apoptotic proteins as well as reducing cytokine release [13]. All these benefits of glucocorticoid may contribute to the recovery of SAKI. In this study, hydrocortisone was shown to be associated with a lower 28-day mortality rate and higher APACHE II and SOFA scores.

AKI is defined with respect to the increase in SCr and decrease in urine output. Our study showed that SCr was obviously decreased and urine volume was significantly increased for patients in the hydrocortisone group. GFR measures the amount of plasma filtered through glomeruli within a given period of time, which is the most widely used indicator of renal function. Reduction in the GFR, secondary to kidney injury, is the hallmark of AKI and results in increased levels of blood urea nitrogen (BUN) and SCr [27]. In line with previous studies by Baylis et al. [28], glucocorticoid was demonstrated to increase GFR in the present study. CysC, a low molecular-weight protein measurable in blood and freely filtered by the glomerulus, is purported to be less affected by age and gender. Some studies have suggested that serum CysC rise with AKI occurs before rise in SCr, leading to evaluation of CysC as an ‘early AKI biomarker’. CysC was shown to detect AKI with an AUC of 0.738 (95% CI, 0.688–0.787) in patients with normal glycemic status and with an AUC of 0.816 (95% CI, 0.738–0.894) in patients with recognized diabetes [29]. In this study, the CysC level was significantly lower for hydrocortisone group at day 7. All these outcomes illustrate that patients’ renal functions could be improved by hydrocortisone.

There are some limitations. First, its single center non-randomized design and the use of non-concurrent control increase the risk of selection bias. To control for the various baseline differences, we performed a PSM adjustment. However, it remains possible that the two groups differed in terms of other factors and PSM may induce a risk of overcorrection by its tendency to adjust many baseline variables in a few rather lopsided matched sets. For instance, the timing of initiation of steroids with the development of AKI is variable and not collected in the study. Moreover, the side effects were not well recorded in this retrospective study. In addition, SCr is not only determined by renal function (excretion), but also by hemodilution (caused by fluid accumulation). It is reported that lower SCr levels caused by fluid overload may underestimate true renal function impairment in critically ill patients [30]. The lack of cumulative fluid balance due to the retrospective nature of the study design may cause information bias. Though some bias may exist in this retrospective study, all recorded potential confounders have been collected and controlled by PSM method to ensure the study quality.

In conclusion, our results indicate that hydrocortisone may improve SAKI patients’ renal functions and decrease the 28-day mortality. However, owing to study limitations, large prospective and randomized controlled trials are required to confirm the efficacy and safety of hydrocortisone for the treatment of patients with SAKI.
